# Impact of Cost-Effective Digital Slide Platform on Grades of Romanian Veterinary Students

**DOI:** 10.3390/vetsci12111064

**Published:** 2025-11-06

**Authors:** Bogdan Gabriel Fuerea, Raluca Ioana Rizac, Nicoleta Ciocîrlie, Teodoru Soare, Manuella Militaru

**Affiliations:** 1 Paraclinical Sciences Department, Faculty of Veterinary Medicine, University of Agronomic Sciences and Veterinary Medicine of Bucharest, 105 Splaiul Independenței, District 5, 050097 Bucharest, Romania; bogdan.fuerea@edu.usamv.ro (B.G.F.); teodoru.soare@fmvb.usamv.ro (T.S.); manuella.militaru@fmvb.usamv.ro (M.M.); 2 Animal Production and Public Health Department, Faculty of Veterinary Medicine, University of Agronomic Sciences and Veterinary Medicine of Bucharest, 105 Splaiul Independenței, District 5, 050097 Bucharest, Romania

**Keywords:** veterinary education, histopathology education, academic performance, e-learning tools, educational technology, affordable technology, remote learning

## Abstract

The global shift toward digital pathology necessitates accessible and cost-effective training tools, posing a significant challenge for resource-constrained veterinary faculties. The objective of this study was to evaluate the academic impact of a cost-effective, in-house-developed digital pathology platform implemented at a Romanian Veterinary Faculty during the 2024–2025 academic year. Grade data from six academic years (2018–2025) were used as historical controls. Statistical analysis, including independent-samples *t*-tests, ANOVA, and multivariable regression, was performed on individual student grade data to assess changes in academic performance and grade distribution. Results demonstrate a statistically significant increase in mean grades and a higher proportion of top-performing students following platform adoption. The 2024–2025 cohort outperformed four of five prior cohorts, showing a marked shift toward higher grades and fewer low grades. We conclude that this low-cost digital pathology platform may significantly enhance student academic outcomes in veterinary pathology education. These findings validate a scalable and sustainable model for improving pedagogical delivery in resource-conscious institutions worldwide. Further longitudinal research is essential to track the long-term sustainability of these academic gains and confirm the platform’s impact across multiple graduating cohorts.

## Simple Summary

This study introduces a low-cost digital slide platform that allows veterinary students in Romania to remotely access high-quality pathology specimens using their own devices. By comparing several years of exam data, we found that students who used it achieved significantly higher grades and demonstrated improved learning outcomes compared to previous student cohorts.

## 1. Introduction

The advancement of veterinary and biotechnology education has been significantly shaped by the ongoing development of quality culture and the adoption of innovative teaching methods. Some studies highlight the integration of quality standards as being essential for continuous improvement in educational outcomes [1,2,3,4]. The shift toward interactive and effective teaching techniques has further enhanced student engagement and practical competencies [5,6,7,8]. Concurrently, the proliferation of e-learning platforms and mobile applications has transformed the educational landscape, offering flexible learning opportunities and increasing student satisfaction in both veterinary and biotechnology fields [9,10,11,12]. This digital transformation is reflected in the positive perceptions of online communication and the use of specialized digital tools in veterinary pathology, such as educational tissue arrays, among students and educators [13,14,15]. Collectively, these developments underscore the importance of embracing digital innovations to meet the evolving needs of modern education and practice.

In today’s digital era, where students have unprecedented access to a vast array of digital data, educational methodologies must evolve to leverage these resources, and consequently digital pathology (DP) has revolutionized education in pathology by transitioning from traditional microscopy to advanced virtual and digital platforms [16,17,18]. This shift, accelerated by technological innovations and societal demands during the COVID-19 pandemic, has enabled the visualization of histopathological and cytopathological slides through whole-slide imaging (WSI) and other digital tools. These platforms offer enhanced accessibility, interactivity, and scalability, allowing students to view slides from multiple angles, annotate regions of interest, and engage in comparative analysis at cellular and subcellular levels [18,19]. Furthermore, DP fosters student-centered learning by integrating tutorials and contextual resources into educational frameworks. In veterinary pathology, these advancements have facilitated more effective teaching methods, addressing challenges associated with conventional practices while improving academic performance and engagement [20,21].

The growing adoption of DP in education has highlighted the need for accessible and cost-effective solutions to address challenges such as limited resources, geographical barriers, and the high costs associated with commercial platforms. While digital tools have demonstrated significant benefits, including enhanced accessibility and improved learning outcomes, many existing solutions remain financially prohibitive for widespread implementation, particularly in resource-constrained settings [22]. Despite the clear benefits of digital pathology, a critical research gap remains in understanding the effectiveness of cost-effective, in-house-developed solutions in student performance, particularly in resource-constrained settings. Addressing this gap is crucial to understanding how such innovations can democratize access to digital pathology and optimize educational outcomes in veterinary pathology [20,21].

The transition to digital pathology fundamentally reshapes the learning dynamic by overcoming the limitations of traditional tools. While glass slides restrict learning to isolated, single-user experiences tied to a physical microscope, WSIs are consistently clearer and offer universal accessibility. This difference is critical: WSI facilitates collaborative teaching by enabling group viewing, real-time annotation, and instant discussion among all students, leading to a richer, more interactive educational exchange.

The primary research question guiding this study is:

- Does the implementation of a cost-effective, in-house-developed digital pathology platform for viewing scanned pathology slides significantly impact student performance in veterinary pathology?

The objectives of this study are threefold: (1) to develop and implement a cost-effective digital platform for viewing scanned pathology slides, addressing the need for accessible and scalable solutions in veterinary education; (2) to evaluate the impact of this platform on student grades in veterinary pathology courses, with a focus on measurable academic outcomes such as mean grades and high achiever rates; and (3) to assess the potential of this platform as a viable and affordable tool for enhancing veterinary pathology education.

## 2. Materials and Methods

### 2.1. Digital Laboratory Hardware and Software Architecture

To ensure replicability and transparency, the following section details the hardware and software components selected for the development and implementation of the cost-effective digital platform for viewing scanned pathology slides.

The digital laboratory platform was designed to support the transition from paper to digital, regarding laboratory data (cases and important slides), which would enable students’ digital access to more learning materials. For this study the main target was enabling students to access digital slides from the comfort of their homes or anywhere else, just by using their own devices (smartphones, tablets, computers, etc.).

For this set-up two purchases were needed: a server with storage and a slide scanner.

As part of the digitization process, four aspects were taken into consideration when choosing the Network Attached Storage (NAS) by Synology^®^ as the main server:

1. Avoid subscriptions and monthly costs—NAS has the capabilities of acting as a web server and also online storage, but also Synology^®^ provides an SSL certificate so that the user can host their own website with an HTTP interface. In this way there is no need to even purchase a domain for hosting the website.
2. Ensure hardware purchased provides many out-of-the-box features—Synology^®^ provides an application to configure the NAS and, although it has numerous in-built functions, the store has free applications to handle any missing functionalities.
3. Ensure the company providing the hardware has experience in doing so, thus increasing the chance of providing a stable piece of equipment, good support and numerous training materials for its usage—Synology^®^ is a long-standing company focused on building NAS for both personal and business use. There are numerous online tutorials for anything the user might need to configure.
4. Provide scalability—the chosen model has 6 bays and also permits the connection of two additional attachments, which would have 4 bays each, meaning it can support up to 14 slots for hard drives. In this case the initial cost would be minimal while permitting the expansion of the storage space with an incremental approach, taking into consideration the fact that technology prices, especially for hard drives, will go down with time.

Regarding software choices, since there is a long-standing open-source option, OpenSeadragon version 5.0.1 was chosen to display digital slides, which narrowed the format of choice for converting the scanned slides to Deep Zoom Images (DZIs). The front-end application was made with Nuxt^®^ UI (as a personal choice due to its low complexity)—Nuxt.js version 3, Nuxt UI version 2.18.7—and the back-end was created with FastAPI version 0.104.1 (using Python version 3.10). Because the NAS provides access to most open-source applications via Docker containers version 3.8, the whole project is created with a single docker project. All software choices were open-source; thus the software cost was zero.

The slide scanner utilized in this study was the Ocus^®^ 40 (Grundium^®^ Oy, Tampere, Finland), a compact, single-slide digital scanner designed for high-precision whole-slide imaging. The Ocus^®^ 40 supports scanning of standard 75 mm × 25 mm glass slides with brightfield illumination and continuous autofocus, achieving optical magnification up to 40×, with digital zoom capabilities for detailed examination at higher effective magnifications [23]. Operated via a web interface, the scanner allows local and remote control from desktops, laptops, or tablets, facilitating flexible integration into digital pathology workflows and enabling students to access high-quality digital slides from any location. Its characteristics mold well to the needs of the Pathology Laboratory.

**Figure 1:**
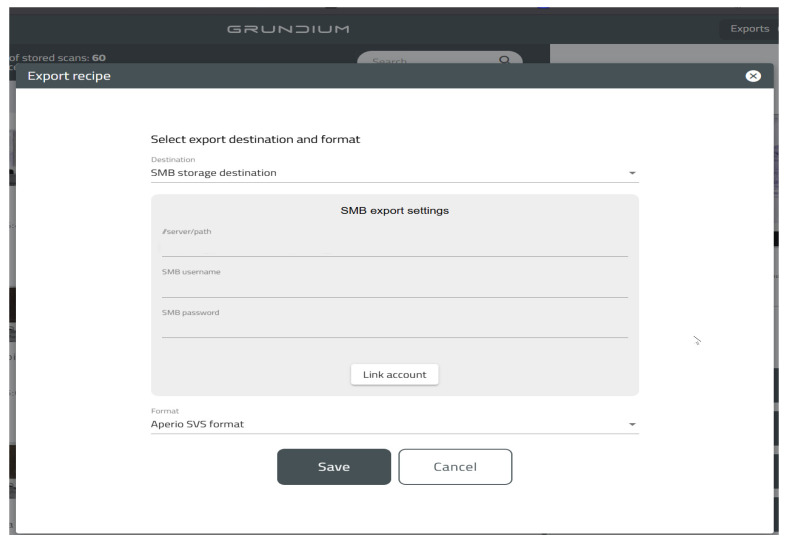
Grundium’s web interface for SMB export settings.

To address challenges with slide storage and viewing compatibility, scanned slides were exported directly to a network-attached storage (NAS) device via the SMB protocol (Figure 1). Exported files were saved in the Aperio^®^ SVS format, chosen for its open, widely supported nature, enabling interoperability across commercial and open-source slide viewers [24,25].

Since the Aperio SVS files were optimized primarily for storage and not fully compatible with the open-source viewer used, a conversion step was implemented. Each slide was converted from Aperio SVS to the Deep Zoom Image (DZI) format using a Python script leveraging the pyvips library, part of the libvips image processing system [26]. The DZI format incorporates multiscale image pyramids composed of tiled images at multiple zoom levels, facilitating efficient navigation and zooming in the digital viewer.

**Figure 2:**
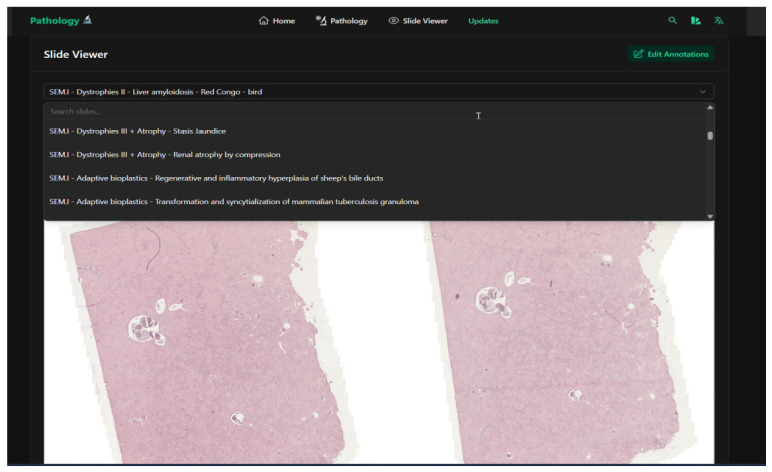
A screenshot from the web application page for viewing digital slides showing the simple dropdown selection (with search functionality) of the existing scanned slides.

The end result was a clean and simple UI, which did not need much computing power to be accessed or served (Figure 2). Instead, for each slide there would be two separate files: Aperio SVS format for archiving and DZI for the students’ platform.

**Figure 3:**
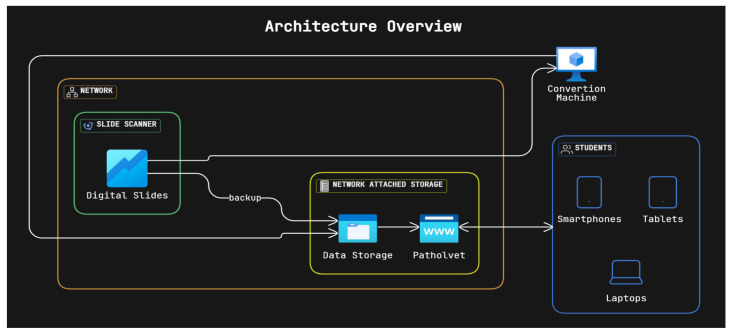
Overview diagram of the current project architecture.

The complete flow (Figure 3) is as follows:

1. The study material slides are chosen and scanned;
2. The digital slides are automatically made available to the teaching staff in Grundium’s website, whereas an exported (Aperio SVS) version is sent to the NAS with two purposes: archiving and conversion;
3. The SVS files are also downloaded on a laptop that can run the python script to convert them to DZI;
4. The same script also allows for synchronization between the laptop and the NAS, uploading the DZI files in the specified path to be picked up by the web application;
5. Once the DZI files for the scanned slide are loaded on the NAS, the Nuxt.js application will automatically make them available to be seen on the website.

### 2.2. Data Collection and Preparation

Raw grade data was retrospectively collected from third-year students studying Pathology in the Faculty of Veterinary Medicine within the University of Agronomic Sciences and Veterinary Medicine of Bucharest across six academic years, spanning from the 2018–2019 to the 2024–2025 academic years. The 2020–2021 academic year was excluded from the analysis due to potential confounding factors related to the global pandemic caused by COVID-19 and exclusive online teaching during this period [27,28].

For the pathology laboratories, the 3rd-year students received theoretical slide explanations supplemented by digital resources such as PowerPoint presentations and magnified images of specimen areas. These materials were accessible through a dedicated website and included in the laboratory textbook. Additionally, students had access to physical slides and microscopes. At the end of each semester, they took a mandatory histopathology examination, with the option to review materials and retake the exam upon failure. This instructional design aimed to develop students’ competencies in slide examination, lesion description, and histopathological diagnosis.

Only grades from the first histopathology exam of the first semester for each academic year were included to obtain consistent and representative performance measures. The dataset comprised failed students (grade 4), passing students (grades 5 to 10), and absentees (marked as “a”). The digital slide platform was implemented solely during the 2024–2025 academic year.

To evaluate year-over-year differences in academic performance while controlling for potential confounders such as variable cohort size and absenteeism, a comprehensive statistical analysis was performed. Descriptive statistics summarized student grades across cohorts. Independent-samples *t*-tests compared performance between the 2024–2025 cohort and prior academic years. Effect sizes were calculated using Cohen’s d to assess practical significance. Additionally, analysis of variance (ANOVA) tested for differences across all academic years, with significant results further analyzed using Tukey’s Honest Significant Difference (HSD) post hoc tests.

**Figure 4:**
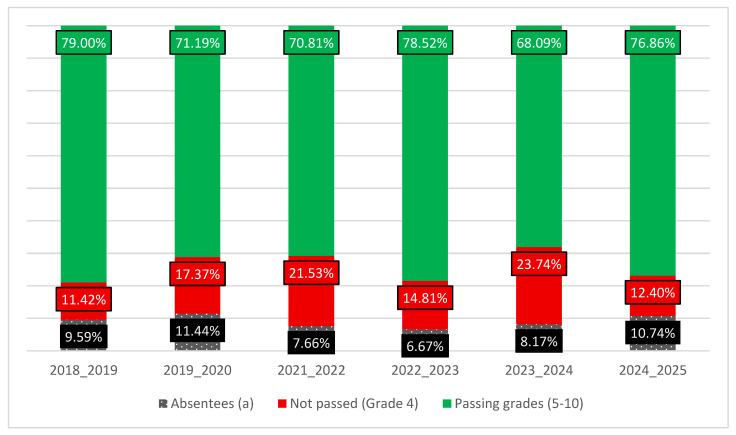
Percentage distribution of exam results (Passing, Not Passing, and Absentee Rates) for the first semester histopathology exam across academic years.

Enrollment numbers, exam participation, and absentee rates varied between academic years; these factors were accounted for in the statistical analyses and are detailed in Section 3 (Figure 4).

### 2.3. Statistical Methodology Explanation

This analysis employed a range of statistical methods to evaluate the impact of the digital platform. These included descriptive statistics, independent-samples *t*-tests, Cohen’s d for effect size, year-over-year change calculations, grade distribution analysis, Analysis of Variance (ANOVA), and post hoc Tukey HSD tests.

Independent-samples *t*-tests were used to compare the mean of the 2024–2025 group with the mean of the combined data from the previous years. This test determines if there is a statistically significant difference between the means of two independent groups.

Cohen’s d was calculated to measure the effect size of the difference in means. It quantifies the magnitude of the difference in standard deviation units, providing an indication of the practical significance of the findings.

ANOVA (Analysis of Variance) was used to compare the means of more than two groups (the different academic years). This test assesses whether there are any statistically significant differences between the group means.

Following the significant ANOVA result, Tukey’s HSD (Tukey’s Honest Significant Difference) test was used for post hoc pairwise comparisons between the means of the different academic years. This test identifies which specific groups have means that are significantly different from each other, while controlling for the family-wise error rate.

To ensure that any observed differences in student outcomes were directly attributable to the digital slide platform and not to yearly fluctuations in demographic or attendance factors, a Multivariable Generalized Linear Model (GLM) was employed. Specifically, a binomial distribution with a logit link function (Logistic Regression) was used to model the odds of a student passing (Grade ≥ 5).

The model included the following variables:

- Independent Variable of Interest: A binary indicator for the Digital Platform Intervention Year (2024_2025).
- Covariates for Statistical Control: The Academic Year Absenteeism Rate and the Total Student Enrollment were included as continuous covariates.

This approach allowed for the estimation of the platform’s effect on student passing rates, net of the concurrent effects of annual changes in absenteeism and enrollment, thus satisfying the requirement to rigorously control for these potential confounding factors.

The results of these analyses provide a comprehensive understanding of the impact of the digital platform on student performance over time.

## 3. Results

### 3.1. Enrollment, Exam Participation, and Absenteeism

The number of third-year pathology students enrolled and those who took the histopathology exam varied across the six academic years analyzed (2018–2019 to 2024–2025), with notable fluctuations in absenteeism (Figure 4).

Across the included academic years, total student enrollment ranged from 209 in 2021–2022 to 270 in 2022–2023. Absentee rates varied between 6.67% (18 of 270 in 2022–2023) and 11.44% (27 of 236 in 2019–2020), with the highest absenteeism observed in 2019–2020. The number of students graded per year ranged from 184 to 252. These fluctuations in enrollment and absenteeism were carefully controlled in the statistical analyses to isolate the impact of the digital slide platform introduced in 2024–2025, and it was concluded that the absenteeism rate and total enrollment were not significant confounding factors in this specific analysis.

A Multivariable Binary Logistic Regression was performed to specifically evaluate the effect of the digital slide platform on the odds of passing (Grade ≥ 5), while statistically controlling for potential confounding factors: the Academic Year Absenteeism Rate and Total Student Enrollment.

The model demonstrated that the introduction of the digital slide platform was associated with a positive increase in the odds of passing, with an Odds Ratio of 1.44 (exp(0.3653)). However, this effect was not statistically significant when controlling for the covariates (β = 0.3653, 95% CI: −0.102 to 0.833, *p* = 0.126).

Furthermore, the control variables, Absenteeism Rate (β = 1.8852, *p* = 0.710) and Total Enrollment (β = 0.0002, *p* = 0.966), were not found to be significant predictors of the odds of passing. This finding validates the robustness of the primary analysis against the influence of annual demographic and attendance fluctuations. For full transparency and reproducibility, the complete dataset and the Python script used for the multivariable analysis are available in the Supplementary Materials.

### 3.2. Academic Performance and Statistical Comparisons

Table 1 summarizes the descriptive statistics of student grades over six academic years, highlighting trends in mean and median grades, passing rates, and the proportion of high achievers. The mean grade fluctuated between 6.29 and 7.34, showing a notable increase in 2024–2025. Median grades remained relatively stable, with a shift from 6.00 to 7.00 in recent years. Passing rates (grades 5–10) varied, reaching a low of 74.15% in 2023–2024 before rising again, while the high achievers (grades 8–10) rate demonstrated a consistent upward trend, peaking at 49.54% in 2024–2025. These data indicate overall improvements in student performance and an increasing proportion of high-achieving students in the most recent academic year.

Independent-samples *t*-tests were conducted to compare the mean grade and high achievers rate of the 2024–2025 cohort with the combined data from the previous five academic years. The results indicated a statistically significant difference in mean grades (*p* = 0.0050) and high achievers rate (*p* = 0.0144).

Cohen’s d analysis for the comparison of the 2024–2025 cohort with the combined data from previous years revealed a large effect size for the difference in mean grades (d = 2.80), suggesting a substantial practical significance of the observed improvement.

Table 2 shows the year-over-year changes in mean grade and high achievers rate. The most notable increase in mean grade was observed between 2023–2024 and 2024–2025 (+0.78), and the largest increase in high achievers rate occurred between 2019–2020 and 2021–2022 (+13.34%), which is irrelevant due to the pandemic year between them, and again between 2023–2024 and 2024–2025 (+13.10%).

**Figure 5:**
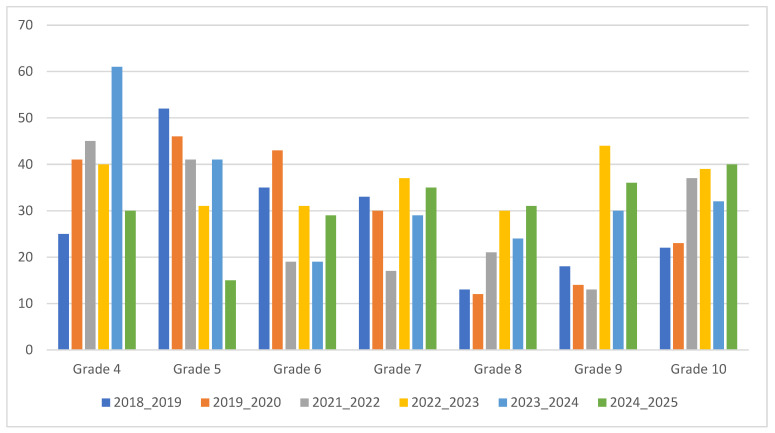
Comparison of number of students for each grade (4–10) for each academic year’s first-semester histopathology exam.

**Figure 6:**
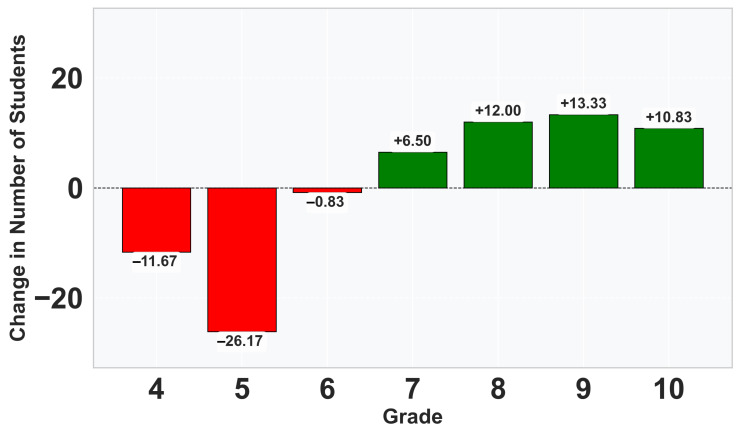
Change in Number of Students per Grade (2024–2025 vs. Historical Average).

Analysis of the grade distribution (Figure 5) in 2024–2025 compared to the historical average of the previous five years showed a decrease in the number of students achieving lower grades (Grade 4: −12.40, Grade 5: −27.20, Grade 6: −0.40) and an increase in the number of students achieving higher grades (Grade 7: +5.80, Grade 8: +11.00, Grade 9: +12.20, Grade 10: +9.40), as represented in Figure 6.

The 2024–2025 cohort demonstrated significant improvements in both average grade and high achievement rates compared to most previous years (Table 3). The most substantial improvement in mean grade was observed when compared to the 2019–2020 cohort (+1.06), while the largest increase in high achievers rate was also seen when compared to 2019–2020 (+26.09%). The effect sizes for these comparisons ranged from negligible to medium.

Analysis of Variance (ANOVA) testing confirms that significant differences exist across all academic years (F = 11.51, *p* < 0.00001). Post hoc Tukey HSD tests, which control for multiple comparisons, demonstrate that the 2024–2025 cohort performed significantly better than four of the five previous years (*p* < 0.01 in all cases). The only exception was 2022–2023, which already showed relatively high performance. The complete Python script and data used for this analysis are provided in the Supplementary Materials.

## 4. Discussion

### 4.1. Superiority of Digital Slides

Learning with digital slides is better than just learning with glass slides. Factors contributing to these outcome include improved accessibility to high-quality histopathology slides, enabling self-paced review outside traditional laboratory hours, thus increasing student engagement and exposure to material, as concluded within the analysis of a feedback form given to 2024–2025 third-year students [29]. While digital slide review complements but does not replace microscope-based examination, it provides a valuable supplemental learning resource.

Enrollment and absenteeism fluctuations across academic years—controlled for in statistical analyses—do not confound the association between platform use and academic improvement, corroborating the robustness of the findings. The exclusion of the 2020–2021 academic year effectively controls for pandemic-related disruptions known to affect learning.

These findings align with prior studies demonstrating the benefits of DP and WSI in veterinary and medical education. Several recent initiatives have applied digital tools to enhance student learning, engagement, and practical skills acquisition [18,20,21,30,31]. Faculty-developed interactive e-learning platforms similarly support improved educational outcomes in related fields [32,33].

### 4.2. Platform Comparison and Technical Improvement

Our results contribute to this body of evidence by demonstrating that a scalable, low-cost digital pathology solution can produce educational impacts comparable to more expensive commercial platforms, particularly important for resource-limited settings.

Technical challenges encountered included certain didactically important slides being thicker than current standards, complicating scanning; however, using multiple slides and iterative scanning overcame this issue. The Grundium web interface lacked role-based access control, requiring additional platforms to secure data integrity by restricting student permissions to view only.

Conversion from Aperio SVS files to DZI format, necessary due to optimization differences, improved viewer performance through faster, multiscale image navigation but introduced additional processing steps. Future workflow optimizations could include direct scanning to DZI or alternative formats to streamline operations.

### 4.3. Strengths, Limitations, and Future Educational Studies

This study has several strengths. The analysis utilizes grade data from multiple academic years, providing a longitudinal perspective on the impact of the digital platform. The use of statistical tests, including *t*-tests, effect size calculations, ANOVA, and post hoc analysis, enhances the rigor of the findings. Furthermore, the focus on a cost-effective, potentially in-house-developed platform adds practical relevance to the study, particularly in resource-constrained educational settings.

The Pathology discipline is characterized by a high degree of standardization, with well-defined rules and procedures governing both course content and laboratory activities. This structured approach ensures that instructional delivery remains consistent across academic years, thereby minimizing variability in teaching methods. As a result, although the study compares outcomes across different cohorts, the potential influence of changes in instructional practice is limited. This consistency strengthens the attribution of observed differences in student performance to the intervention under investigation, rather than to fluctuations in teaching methodology.

However, there are also limitations to consider. This study employs a pre- and post-implementation design without a concurrent control group. While the significant improvements observed in the year following the platform’s introduction suggest a causal relationship, other factors that may have changed between academic years (e.g., generational cohort effects and external factors affecting student learning) cannot be entirely ruled out. The study is also specific to the student population and the particular veterinary pathology course under investigation, and the generalizability of these findings to other contexts may need further exploration.

The positive outcomes support wider adoption of digital pathology platforms within veterinary curricula. Providing students with convenient, anytime access to digital slides enhances engagement and comprehension, which is critical in developing histopathological diagnostic competence. The cost-effectiveness and scalability of the platform further recommend its use in resource-constrained educational environments.

Enhancements such as integrating interactive 2D/3D visualizations of pathological structures could further support spatial reasoning and conceptual understanding, consistent with evidence from analogous domains [34]. Additionally, collaborative initiatives, such as those linking veterinary faculties in creating shared digital slide repositories [35], offer promising directions to expand access and resources.

By incorporating these elements and adapting the teaching methods (e.g., focusing on interactiveness [13]), digital pathology platforms can further enhance the learning experience and prepare students for the challenges of modern veterinary practice.

Based on the findings and limitations of this study, several avenues for future research can be identified. Investigating the long-term impact of the platform on student learning and retention of knowledge would also be valuable. Gathering qualitative data through additional surveys or interviews to explore students’ perceptions of the platform’s usability, its impact on their learning experience, and specific features they find most beneficial could provide richer insights. Furthermore, comparing the effectiveness of different features within the platform or comparing this cost-effective platform with commercially available systems could offer valuable information for educators and developers. Also, exploring the applicability of this approach in other disciplines within veterinary medicine or other fields of study could broaden the impact of this research.

Khetsha [36] explains that teaching agricultural subjects works best when students actively build their own understanding and reflect on what they learn. This “learning by doing and thinking” approach fits well with using new tools like digital slides to make learning veterinary cytology and histology more engaging. The study also points out that teachers need proper training and support to be able to use these modern, technology-based teaching methods effectively.

An important direction for future research involves a comprehensive evaluation of how the integration of digital platforms influences final summative assessment outcomes in pathology. Investigating this relationship could provide valuable insights into whether digital tools enhance students’ mastery of core concepts or merely facilitate access to learning resources. Additionally, further studies should explore the extent to which digital platforms affect student motivation and satisfaction within veterinary pathology, considering both the perceived effectiveness and personal engagement with online learning modalities [20]. Equally significant is the need to assess the impact of these technological advancements on teaching staff, including changes in pedagogical strategies, workload, and professional satisfaction. Research indicates that adoption rates of digital technologies vary across sectors, with some studies highlighting gradual yet significant uptake among stakeholders in traditionally less digitized environments, such as greenhouse management [37]. Understanding such adoption dynamics can inform strategies to enhance technology uptake among teaching staff. Such research would offer a holistic understanding of the transformative effects of digitalization in veterinary pathology education and inform best practices for future implementation.

## 5. Conclusions

This study proposes that an in-house digital pathology platform can greatly improve veterinary pathology education by making learning more engaging and effective. Its accessibility and affordability make it a valuable tool for expanding learning opportunities, especially in resource-limited settings.

## Figures and Tables

**Table 1:** Descriptive statistics for student grades across the academic years.

Academic Year	Mean Grade	Median Grade	Passing Rate	High Achievers Rate
2018–2019	6.50	6.00	87.37%	26.77%
2019–2020	6.29	6.00	80.38%	23.44%
2021–2022	6.60	6.00	76.68%	36.79%
2022–2023	7.09	7.00	84.13%	44.84%
2023–2024	6.56	6.00	74.15%	36.44%
2024–2025	7.34	7.00	86.11%	49.54%

**Table 2:** Year-over-year changes in mean grade and high achievers rate.

Comparison	Mean Grade Change	High Achievers Rate
2018–2019 to 2019–2020	−0.21	−3.32%
2019–2020 to 2021–2022	+0.31	+13.34%
2021–2022 to 2022–2023	+0.49	+8.05%
2022–2023 to 2023–2024	−0.53	−8.40%
2023–2024 to 2024–2025	+0.78	+13.10%

**Table 3:** Target study year comparison to previous years.

2024–2025 Compared to:	Mean Grade (Δ, %)	Effect Size (Cohen’s d)	Estimated *p*-Value	High Achievers (Δ, %)	Passing Rate (Δ)
2018–2019	+0.84 (+12.96%)	0.42 (Small)	0.0210 (Significant)	+22.77% (+85.06%)	−1.26%
2019–2020	+1.06 (+16.79%)	0.53 (Medium)	0.0038 (Significant)	+26.09% (+111.29%)	+5.73%
2021–2022	+0.75 (+11.32%)	0.37 (Small)	0.0409 (Significant)	+12.75% (+34.66%)	+9.43%
2022–2023	+0.26 (+3.60%)	0.13 (Negligible)	0.4845 (Not significant)	+4.70% (+10.47%)	+1.98%
2023–2024	+0.78 (+11.94%)	0.39 (Small)	0.0319 (Significant)	+13.10% (+35.94%)	+11.96%

## Data Availability

The original contributions presented in this study are included in the article. Further inquiries can be directed to the corresponding authors.

## Supplementary Materials

The following supporting information can be downloaded at: https://www.mdpi.com/article/10.3390/vetsci12111064/s1.
